# Evaluation and Modeling of Polylactide Photodegradation under Ultraviolet Irradiation: Bio-Based Polyester Photolysis Mechanism

**DOI:** 10.3390/polym16070985

**Published:** 2024-04-04

**Authors:** Sergey Lomakin, Yurii Mikheev, Sergey Usachev, Svetlana Rogovina, Lubov Zhorina, Evgeniya Perepelitsina, Irina Levina, Olga Kuznetsova, Natalia Shilkina, Alexey Iordanskii, Alexander Berlin

**Affiliations:** 1 N. N. Semenov Federal Research Center for Chemical Physics Academy of Science, 119991 Moscow, Russia; usachevsv@inbox.ru (S.U.); s.rogovina@mail.ru (S.R.); 30111948l@bk.ru (L.Z.); 123zzz321@inbox.ru (O.K.); tashi05@list.ru (N.S.); berlin@chph.ras.ru (A.B.); 2 Emanuel Institute of Biochemical Physics, Russian Academy of Sciences, 119334 Moscow, Russia; mik@sky.chph.ras.ru (Y.M.); iilevina@inbox.ru (I.L.); 3 Federal State Research Center for Chemical Physics and Medical Chemistry, Russian Academy of Sciences, 142432 Chernogolovka, Russia; oligo2019@icp.ac.ru

**Keywords:** polylactide, Differential Scanning Calorimetry, Gel Permeation Chromatography, IR Spectroscopy, NMR Spectroscopy, kinetics, UV radiation

## Abstract

In our study, we investigated the accelerated aging process of PLA under 253.7 nm UV-C irradiation with the use of the GPC, NMR, FTIR, and DSC methods and formal kinetic analysis. The results of GPC and DSC indicated a significant degree of destructive changes in the PLA macromolecules, while spectroscopic methods NMR and FTIR showed maintenance of the PLA main structural elements even after a long time of UV exposure. In addition to that, the GPC method displayed the formation of a high molecular weight fraction starting from 24 h of irradiation, and an increase in its content after 144 h of irradiation. It has been shown for the first time that a distinctive feature of prolonged UV exposure is the occurrence of intra- and intermolecular radical recombination reactions, leading to the formation of a high molecular weight fraction of PLA decomposition products. This causes the observed slowdown of the photolysis process. It was concluded that photolysis of PLA is a complex physicochemical process, the mechanism of which depends on morphological changes in the solid phase of the polymer under UV radiation.

## 1. Introduction

In the frame of the bio-circular economy [1,2], the replacement of petroleum-based plastics with eco-friendly biodegradable polymers such as polylactides (PLAs), polyalkanoates (PHAs), poly(ε-caprolactone) (PCL), and others, as well as the assessment of their decomposition mechanisms and exploitation lifetimes [3], are the key challenges in environmental management, packaging, and biomedicine. As is well-known, PLA is a linear aliphatic polyester produced from the *L* and *D* isomers of lactic acid formed during the fermentation of the natural raw materials [4,5]. Polylactides’ family, as the main commercial product on the market comprising biodegradable materials, is broadly implemented in many areas such as food packaging, eco-friendly materials design, drug delivery vehicles exploitation, and others [6,7].

Polylactides with diverse chemical structures, crystallinity, and morphology are used as biodegradable, biocompatible, and sustainable plastics. They are successfully utilized for biomedical implant engineering [8], drug delivery platform elaboration [9], separation/filtration membrane design [10], active packaging implementation [11], eco-friendly safety measure applications [12], and many others. PLA mechanical behavior can be compared with conventional synthetic polymers like PP (polypropylene), PS (polystyrene), and PVC (polyvinylchloride), which were suitably summarized in a comprehensive work [13]. Furthermore, tensile and flexural strengths for PLA have higher values than PVC and PP. Additionally, under low and ambient temperatures, the bio-polyester given shows an appropriate thermal destruction stability with a wide melt-processing window [14] and efficient oil/water separation capability [15].

As the basic factor of climatic change, CO_2_ gas emission during PLA biodegradation is approximately 1.60 kg/kg, which is less than for PP (~1.85 kg/kg) and essentially less than for PS (~2.74 kg/kg), PET (~4.15 kg/kg), and nylon (~7.15 kg/kg) biodegradation [13]. Besides, the substitution of the above synthetic polymers with PLA produced on the basis of agricultural substrates can potentially lead to a significant decrease in the emission of greenhouse gases [13]. Concerning the environmental advances of PLA, Vink et al. have very recently reported [16] that the amount of fossil energy being spent on PLA production is 25–55% less than the energy required for producing petrol-based plastics.

A significant number of works are presented in the literature that have explored the impact of UV radiation on kinetics and the mechanism of PLA photodegradation, where the polyester occurs as a homopolymer, blends, and composites [17,18,19,20,21,22]. These works were devoted to the study of molecular mass evolution, analytical assessment of the chemical composition comprising the intermediate and final products of photodegradation, the analysis of changes in the morphology and crystallinity, and tests of mechanical behavior.

However, a number of previous studies devoted to photodegradation in the spectral wavelength range above 300 nm have been carried out where the irradiation energy is too small to disrupt the covalent bonds in the PLA backbone [23]. As is widely acknowledged, PLA items are generally applicable in biomedicine, packaging, and environmental exposition, where UV emission occurs at 254 nm for sterilization [24] and weathering explorations via European standard ISO 4892-2:2013 [25,26].

The principal inconveniences of PLA’s usage in biomedicine comprise its molecular hydrophobicity, which manifests itself in the low concentration of functional groups involved in cell adhesion. Poor hydrophilicity constrains the elaboration of scaffolds and conduits [27]. Moreover, the biodegradation of PLA occurs under relatively harsh conditions when the samples are composted or exposed to seawater [28]. Therefore, polyester utilization is challenging, with certain problems. One of the ways to tailor this concern is the blending of PLA with natural polysaccharides like cellulose and starch, which are lightly released into aqueous media [29,30]. After the transition of the polysaccharide from the blended matrix into soil or water, the integrity of the blend is significantly impaired, the inherent surface of PLA being available for enzymatic attack is increased, and the rate of biodegradation of the polyester is essentially enhanced. An alternative way to initiate PLA decomposition is its photodegradation under UV irradiation, which was explored in a series of pioneering works by Ikada and Ashida [31,32]. Wherein, the authors put forward the hypothesis that the photodegradation of PLA complies with the Norrish II mechanism. These studies led to the conclusion that UV exposure is highly effective in polymer waste processing and the design of plastics with a controlled service life.

**Figure 1:**
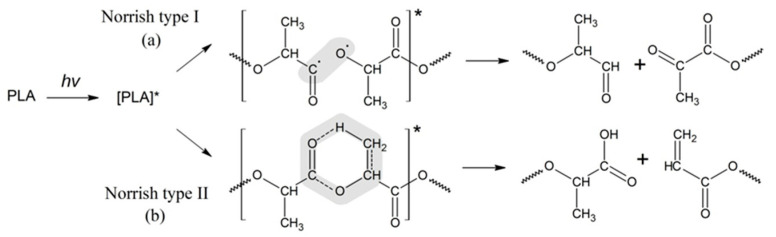
Schemes of the PLA photodegradation process according to the Norrish I and Norrish II mechanisms (* refers to the excited state of the carbonyl groups in PLA as a result of UV irradiation).

Further study of PLA photolysis was continued in a number of publications [20,21,23,33]. In particular, Tsuji et al. [34] investigated the impact of PLA morphology on the rate of photodegradation. It was found that photodegradation in amorphous regions of PLA proceeds faster than in crystalline ones, and the cleavage of ester bonds in macromolecules occurs via the Norrish II mechanism as well. In a series of comprehensive publications, the experts have advanced the basic mechanism of PLA photodegradation determined as a combination of the Norrish I and Norrish II types of polymer chain scission [17,35]. The scheme of the PLA photodegradation process according to the Norrish I and Norrish II mechanisms is presented in Figure 1.

Meanwhile, a series of follow-up experiments have shown that the real way of PLA decomposition is still under consideration. So, in the above publications, Ikada has claimed that the exposure of PLA to UV irradiation leads to the Norrish II-type mechanism mostly, namely that the PLA backbone absorbs a UV photon with sufficient energy to break the C-O bonds [31]. Alternatively, Olewnik-Kruszkowska and coauthors [23] have proposed another way of degradation where acetic anhydride is involved in a set of radical reactions.

For the Norrish I mechanism of PLA photodegradation, the reaction is realized through the homolytic breaking of the bond C-O-R belonging to the carboxylic group, which results in aldehyde and diketone derivatives, while the Norrish II type of degradation (Figure 1b) occurs via the formation of a six-membered intermediate cycle. However, the use of only one of the proposed types of mechanisms does not allow for a quantitative assessment of the kinetics of PLA photodegradation.

The analysis of the above situation allows the authors to suggest that the structural evolution of PLA exposed to UV radiation proceeds in a more complex fashion, involving a combination of the Norrish reactions. Besides, it is appropriate to consider the role of intermolecular interactions of the macro-radicals, leading to branched and cross-linked structures. This paper focused on experimental examination with the DSC, NMR, FTIR, and GPC techniques, the kinetic aspect of photodegradation, and modeling the decomposition of PLA molecules. The outcomes of this presentation may have the potential to advance the development of PLA-based materials exploited under UV impact, making an essential contribution to the evaluation of their photolysis resistance in biomedicine and environmental sustainability.

## 2. Materials and Methods

### 2.1. Materials

Polylactide (PLA) grade PLA 4043D (Nature Works, Minnetonka, MN, USA (M_w_ = 1.3 × 10^5^ g/mol, T_m_ = 163 °C) was used in this work.

### 2.2. Preparation of Samples

Films were prepared by dissolving PLA in chloroform, which was mixed with mechanical stirring. The solutions were poured onto Petri dishes. The solvent was removed by slow evaporation at room temperature and then at 50 °C until a constant weight. The thickness of the films was 0.2–0.3 mm.

### 2.3. UV Irradiation

The effect of ultraviolet (UV) radiation on the PLA films was studied at a wavelength of 253.7 nm and the lamp power (4 Philips TUV lamps) was 11 W. During irradiation, the film samples were placed in a chamber where they were exposed to UV radiation. In order to maintain uniform exposure, the samples mounted on a holder rotated uniformly inside the chamber at a speed of 6 rpm. The exposure time was 2, 5, 24, and 144 h.

### 2.4. Gel Permeation Chromatography (GPC)

The molecular mass characteristics of the samples were determined by means of Gel Permeation Chromatography (GPC) on a liquid chromatograph by Waters (Milford, MA, USA) equipped with refractometric and UV detectors. Eluent—tetrahydrofuran, elution rate 1 mL/min, column temperature 35 °C, refractometer temperature 45 °C. Polymer samples were dissolved in THF and the solution was filtered through a PTFE filter Anatop 25 (Whatman) supplied by Merck KGaA, Darmstadt, Germany. For measurements, two PL gel columns connected in series, and MIXED-C 5 µm were used. The average molecular weight (M_w_) of the PLA samples was calculated using a calibration curve, obtained by means of polystyrene standards with a M_w_ from 589 to 3.7 × 10^6^ Da.

### 2.5. Differential Scanning Calorimetry (DSC)

The thermophysical characteristics of the PLA samples exposed to UV irradiation were studied using the DSC method on a DSC-204 F1 (NETZSCH-Gerätebau GmbH, Germany, Selb, Bavaria.) calorimeter at a heating rate of 10 K/min in an inert atmosphere of Ar in the temperature range of 25–200 °C. The use of a repeating heating–cooling mode in DSC studies of polymers in order to remove the “prehistory” of their production is generally accepted. However, in this work, DSC studies were carried out in one-stage mode without reheating, since we intended to characterize the primary morphology of UV-irradiated PLA samples, rather than “erase their thermal memory” or “thermodynamically balance” their initial structure.

The degree of crystallinity of the PLA samples, χ%, was calculated by this equation:

$$\chi =\frac{\Delta H_{m}-\Delta H_{cc}}{\Delta H_{m}^{100}}$$

where $\Delta H_{m}$—enthalpy of melting, $\Delta H_{cc}$—enthalpy of crystallization (enthalpy of “cold” crystallization) and $\Delta H_{m}^{100}$—the theoretical value of the of 100% crystalline poly(L-lactide) melting enthalpy (93.6 J/g) [36].

### 2.6. NMR Spectrometry

^1^H NMR spectra (500.18 MHz) were recorded on an Avance III 500 spectrometer (Bruker, Germany, Karlsruhe) in CDCl_3_.

### 2.7. FTIR Spectrometry

The infrared spectra of the PLA before and after the UV-radiation had acted on it were acquired with the aid of a Bruker Tensor 27 IR Fourier spectrometer with an ATR PIKE Miracles™ accessory (PIKE Technologies, Madison, WI, USA) equipped with a germanium (Ge) crystal. The IR spectra were recorded in the range of 4000–700 cm^−1^ with a resolution of 4 cm^−1^ and averaging over 32 successive scans. The spectra were normalized after the baseline correction of the entire spectrum with the use of the Min–Max Normalization method by OPUS 7.5 software.

## 3. Results and Discussion

Our study aimed to investigate the accelerated aging process of PLA thin films under 253.7 nm UV-C irradiation for an initial 24 h and continuous irradiation for 144 h with the use of the GPC, NMR, FTIR, and DSC methods. The results of analyses using the GPC and DSC methods indicated a significant degree of destructive changes in PLA macromolecules, while the spectroscopic methods NMR and FTIR showed the maintenance of the PLA main structural elements even after a long time of UV exposure. In addition to that, the GPC method displayed the formation of a high molecular weight fraction starting from 24 h of irradiation, with an increase in its content after 144 h of irradiation.

### 3.1. Gel Permeation Chromatography (GPC)

**Figure 2:**
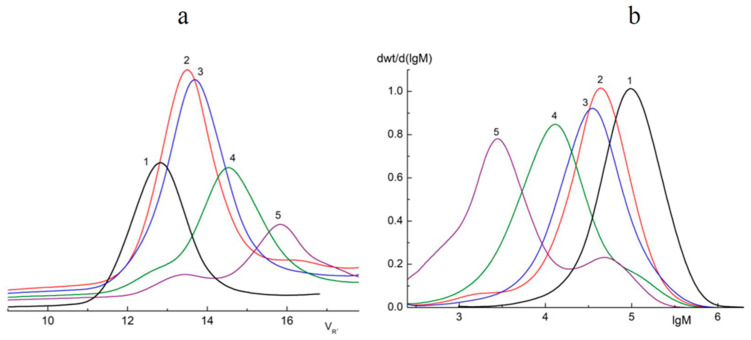
Chromatograms (**a**) and M_w_ curves (**b**) of the samples of initial PLA (1) and PLA after 2 (2), 5 (3), 24 (4), and 144 (5) hours of UV irradiation.

The GPC method was used in order to determine the molecular weight distribution (M_w_) of oligomers formed during the photodegradation of PLA. The results obtained are presented in Figure 2 and Table 1.

From the data presented in Table 2, a sharp decrease in M_w_ just after 2 h of UV irradiation is clearly seen. However, after 5 h of UV exposure, there was a distinct slowdown in the process. Furthermore, PLA samples exposed to irradiation for 24 and 144 h, in addition to the main photodegradation fractions M_w_ = 12,590 and 2818, also contained fractions with larger values of M_w_* = 120,220 and 48,980, respectively (Table 1). In order to calculate the percentage of the main fractions obtained after 24 and 144 h of UV irradiation, the deconvolution of the asymmetric peaks (M_w_ and M_w_*) presented in the curves (Figure 2b) was carried by the Fraser–Suzuki algorithm using NETZSCH Peak Separation version 2006.01 software [37,38,39] (Supplementary Figure S1).

Araujo et al. [40] had previously reported similar results in a GPC study of PLA 4042D photodegradation under UV with λ = 365 nm. Two peaks on the chromatograms corresponding to the low and high molecular mass fractions of PLA were detected. We suggested that the accumulation of high molecular fractions under long-term UV irradiation could be explained by intermolecular reactions of PLA macroradicals recombining.

Thus, the GPC results of the PLA photodegradation products suggest a more complex mechanism of photolysis than the mechanism presented previously in the works of Ikada [31,32] (Figure 1).

### 3.2. Kinetics of PLA Photodegradation

**Figure 3:**
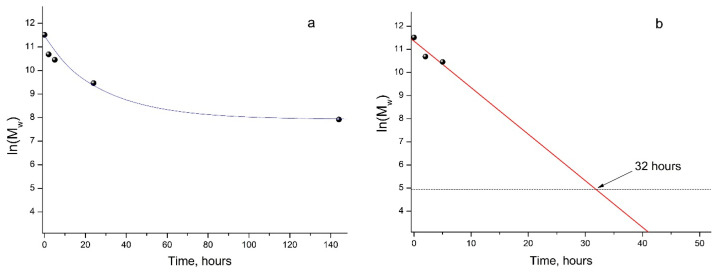
Kinetic curve of PLA photodegradation (**a**); linear dependence of ln(M_w_) vs. time (**b**) according to regression analysis data (Supplementary Figure S2).

As mentioned above, the GPC results showed a steady decrease in the M_w_ of PLA samples under UV irradiation (Figure 2b), which made it possible to provide a formal kinetic analysis of the process. Data presented in Figure 3 (a kinetic curve) clearly show the nonlinear dependence of ln(M_w_) on the PLA photodegradation time.

At the same time, during the first 5 h of UV exposure, a quasi-linear dependence of ln(M_w_) values on time is observed (Figure 3, straight line b).

This allowed us to assume the formal first-order kinetic mechanism of PLA photodegradation:

(1) $$-\frac{dM_{w}}{dt}=k_{fd}\times M_{w},\;\ln M_{w}=-k_{fd}\times t+A$$

The value of the PLA photodegradation rate constant *k_fd_* = 0.20117 h^−1^ = 5.6 × 10^−5^ c^−1^ was calculated by the use of linear regression analysis (Supplementary Figure S2).

**Figure 4:**
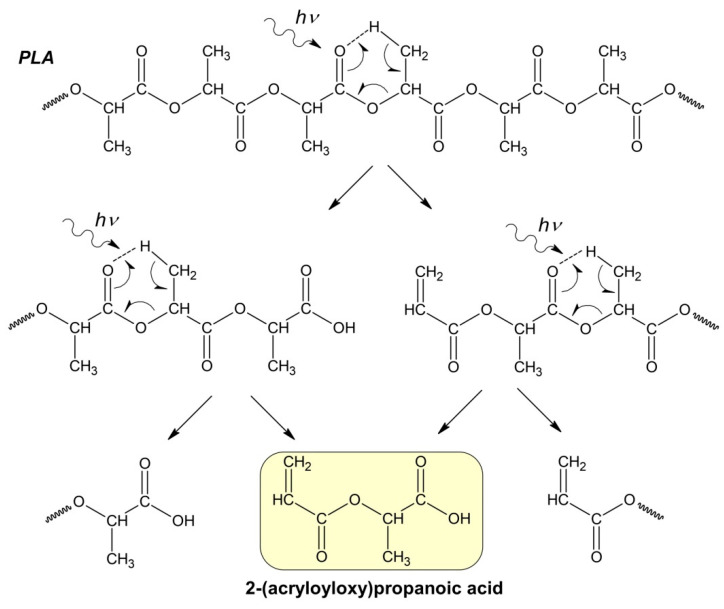
Hypothetical mechanism of PLA photodegradation (Norrish II) according to the formal first-order kinetic equation.

Apparently, at the initial stage of photodegradation (first 5 h), the PLA macromolecules break up along ester bonds according to the Norrish II mechanism, forming oligomeric fractions containing terminal carboxyl and vinyl groups (Figure 4).

One can suppose that PLA photodegradation proceeds in one stage (first order) according to the Norrish II mechanism while maintaining the flexibility of PLA macromolecules and the equiprobable formation of intermediate six-membered transition states in the monomer units (Figure 1) throughout the entire interval of UV irradiation time.

In this case, the PLA photodegradation according to the Norrish II mechanism will eventually result in a low molecular weight ultimate product of 2-(acryloyloxy)propanoic acid with M_w_ = 144 g/mol (ln(M_w_) = 4.96). Taking into account the calculated value of the reaction rate constant k*_fd_* = 5.6 × 10^−5^ c^−1^, it can be determined that the initial PLA with M_w_ = 13.27 × 10^5^ will decompose completely to the ultimate product of 2-(acryloyloxyloide) with M_w_ = 144 in 32 h (Figure 3b).

Similar data were obtained when studying the degradation of amorphous PLA under UV irradiation at 300 and 365 nm, where the almost complete destruction of the polymer in 12 h was explained by the absence of a PLA crystalline phase [31]. This is probably why, with longer UV irradiation of PLA, a decline in the rate of photodegradation was observed (Figure 3, curve a). This fact may be associated with the increasing role of recombination reactions of primary PLA macroradicals by restoring the original bonds, as well as with the simultaneous occurrence of intermolecular recombination reactions of macroradicals of neighboring chains, which is confirmed by the GPC data (Table 1). These reactions reduce the overall rate of the PLA photodegradation process, as a result of which a nonlinear dependence of ln(M_w_) vs. time is observed (Figure 3a). An additional factor slowing down the process is the presence of a hard crystalline phase in the PLA matrix.

It can be assumed that the crystalline fragments of PLA macrochains are under various strains, preventing the formation of the six-membered transition states necessary for the implementation of photodegradation by the Norrish II mechanism. That is why the decomposition of PLA under the influence of UV irradiation by the Norrish I mechanism becomes preferable.

In order to determine the content of the crystalline phase in PLA samples exposed to UV radiation, we conducted DSC experiments.

### 3.3. Differential Scanning Calorimetry (DSC)

**Figure 5:**
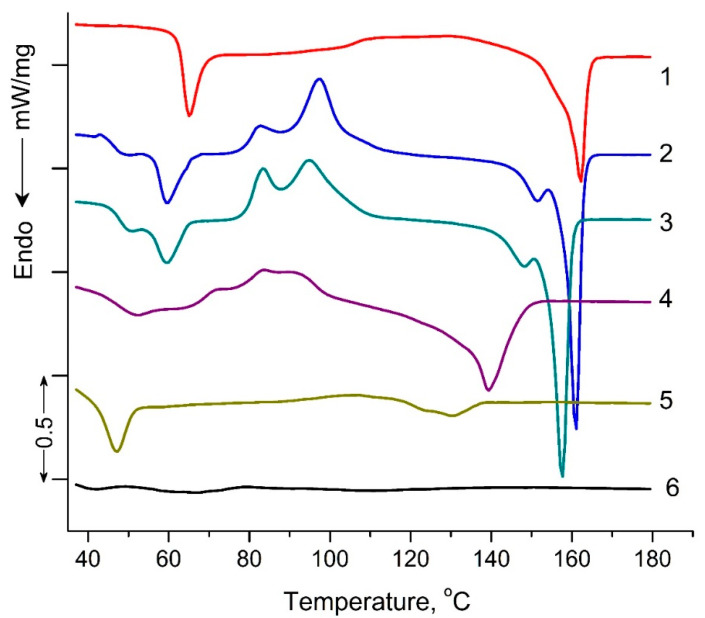
DSC heat flow curves of pristine PLA (1), PLA UV irradiated for: 2 (2), 5 (3), 24 (4), 48 (5), 144 (6) hours.

Figure 5 shows DSC thermograms of PLA samples exposed to UV irradiation. The characteristic DSC parameters include: glass transition *T*_g_, peak temperatures for cold crystallization *T*_cc_ and melting *T*_m_, peak temperature of enthalpy relaxation *T*_r_, characteristic enthalpies associated with cold crystallization Δ*H*_cc_ and melting Δ*H*_m_, and degree of crystallization (χ), and are summarized in Table 2. Small “relaxation” effects in PLA samples (except for PLA exposed to UV irradiation for 144 h) illustrated by endothermic heat capacity peaks (*T*_r_) can be seen at temperatures above the *T*_g_ (Figure 5). These peaks characterize the phenomenon of enthalpy relaxation, which is associated with the mobility and the recovery of PLA chains to their thermodynamic equilibrium status at a temperature above *T*_g_. 

As can be seen from Figure 5, a transition from a glassy to a highly elastic state was observed for all PLA samples in the temperature range of 63.5–35.6 °C. At the same time, the glass transition temperature (T_g_) values decreased as the UV exposure time of the samples increased from 63.5 °C for the original PLA sample to 35.6 °C for the PLA sample UV-irradiated for 144 h (Table 2). At temperatures above 90 °C, for all PLA compositions exposed to UV irradiation, except for the PLA sample UV-irradiated for 48 and 144 h, the exothermic effect of “cold crystallization” was observed (Table 2). It is noteworthy that for PLA samples exposed to UV irradiation for 2, 5, and 24 h, a double exo-peak of cold crystallization is observed in Figure 5, which is characteristic of the formation of two different crystalline PLA forms.

The most interesting research results are the data on the melting temperatures and degrees of PLA sample crystallinity (Table 2). For the PLA samples exposed to 2, 5, 24, and 48 h of UV irradiation, similar to cold crystallization, double melting endo-peaks are observed. These peaks are characteristic of *α*-ordered (orthorhombic) and *α*′-limit disordered (hexagonal) crystalline forms of PLA. At the same time, the degree of crystallinity for PLA samples exposed to 2, 5, 24, and 48 h of UV irradiation decreased from 20.4 to 5.1%, and the sample exposed to UV irradiation for 144 h became completely amorphous (Table 2).

The DSC results showed significant structural changes in UV-irradiated PLA samples compared to the original polylactide. The most pronounced changes in thermodynamic parameters were observed at the maximum time of UV exposure of PLA (144 h), which suggests qualitative structural transformations of the sample. In order to specify the structural changes in the irradiated PLA samples, NMR and FTIR methods were used in this work.

### 3.4. NMR Analysis

**Figure 6:**
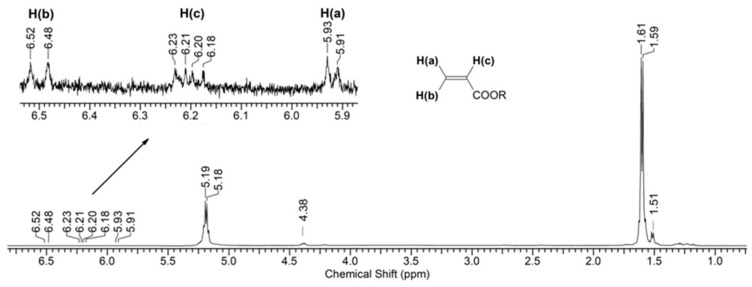
^1^H NMR spectrum of PLA after 144 h of UV irradiation.

Figure 6 shows the (^1^H) NMR spectrum of a PLA sample after 144 h of UV irradiation. As can be seen from the spectral data, the main signals are the multiplets corresponding to the protons of the initial PLA: (*δ*, ppm) 1.60 (3H, CH_3_) and 5.19 (1H, CH) (Supplementary Figure S3). Moreover, the spectrum also contains minor signals of the terminal group of lactic acid (-CH(OH)CH_3_): (*δ*, ppm) 1.51 (3H, CH_3_) and 4.38 (1H, CH) (Supplementary Figure S4). Also, in the spectrum one can observe weak signals that relate to the double bond of the C=C-C(O)O- group (^2^*J*_H(a)H(b)_ = 1.5 Hz, ^3^*J*_H(b)H(c)_ = 17.0 Hz, and ^3^*J*_H(a)H(c)_ = 10.0 Hz) (Supplementary Figure S5). However, their intensity indicates an insignificant concentration of double bonds in the sample.

**Figure 7:**
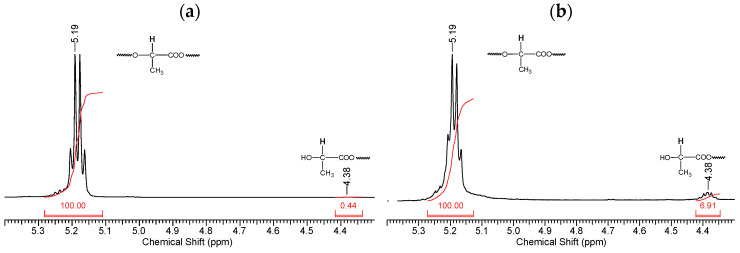
Fragments of NMR spectra related to the chemical shifts of protons (5.19 ppm) of the PLA polymer chain at the tertiary carbon atom and terminal CH_3_ groups (4.38 ppm): (**a**) initial PLA, (**b**) PLA after UV irradiation (144 h).

Fragments of the NMR spectrum of pristine PLA and PLA after 144 h of UV irradiation show an increase in the number of -CH(OH)CH_3_ end groups (Figure 7b). This is evidenced by the ratio of the integral intensities of protons belonging to the polymer chain (5.19 ppm) and terminal groups (4.38 ppm).

Thus, based on the NMR data (Figure 6 and Figure 7), we can conclude that PLA macromolecules decompose in at least two directions under UV exposure. As a result of one of them, double C=C bonds are formed with the participation of CH_3_ groups, which may be associated with the photodissociation of PLA according to the Norrish II mechanism (Figure 1). However, the very low intensity of signals from vinyl protons in the NMR spectrum (Figure 6) indicates the weakly expressed nature of this mechanism. Another direction of PLA photolysis is the destruction of the ester bond without the participation of the CH_3_ group, which can be explained by hydrolytic processes involving traces of atmospheric water. This direction was studied in the work of Copinet et al. [41], when UV irradiation of PLA was carried out under conditions of high humidity. It was shown that in this case, hydrolytic processes are significantly intensified. NMR analysis also showed that the main structural fragments of PLA were preserved even in the case of the formation of oligomers identified using GPC. Similar characteristics of the structural changes in PLA were found by FTIR analysis.

### 3.5. FTIR Analysis

**Figure 8:**
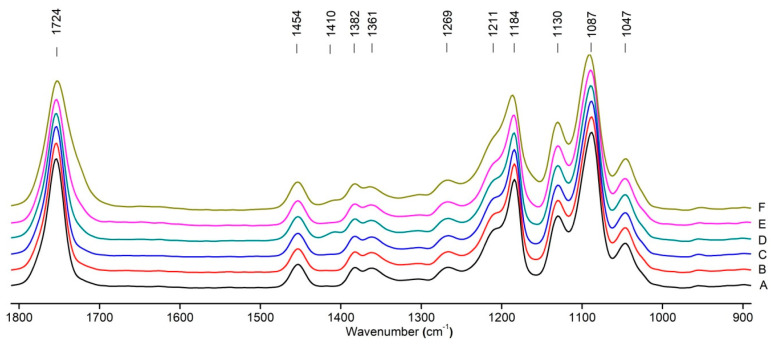
FTIR spectra of pristine PLA (A) and after UV irradiation: (B) (2), (C) (5), (D), (24), (E) (48), (F), (144) hours.

Figure 8 shows the IR spectra of PLA samples at different times of UV irradiation. The main characteristic frequencies of the PLA spectra after exposure to UV irradiation are given in Table 3.

Significant changes can be observed in the 1235–1160 cm^−1^ area of asymmetric vibrations of the C-C(O)-O group [42]. In this region, a broadening of the absorption bands is observed, which is especially noticeable for the sample exposed to UV irradiation for 144 h. One can also note the broadening of the band at 1754 cm^−1^ related to the carbonyl group C=O [42,43,44] due to the formation of a poorly resolved peak at 1724 cm^−1^. Apparently, this peak belongs to the carbonyl of the ester group, the spatial configuration and environment of which differs from the carbonyl in the original *L*-PLA. Meanwhile, the intensity of this peak increases with the time of UV irradiation. To quantify the obtained FTIR data, peak separation (deconvolution) of the peaks in the region of 1850–1680 cm^−1^ belonging to the C=O stretching vibrations of ester groups was carried out. Deconvolution of the overlapping FTIR peaks at 1724 and 1754 cm^−1^ was performed using NETZSCH Peak Separation 2006.01 program employing the GAUSS algorithm for symmetric signals [39] (Table 4).

**Figure 9:**
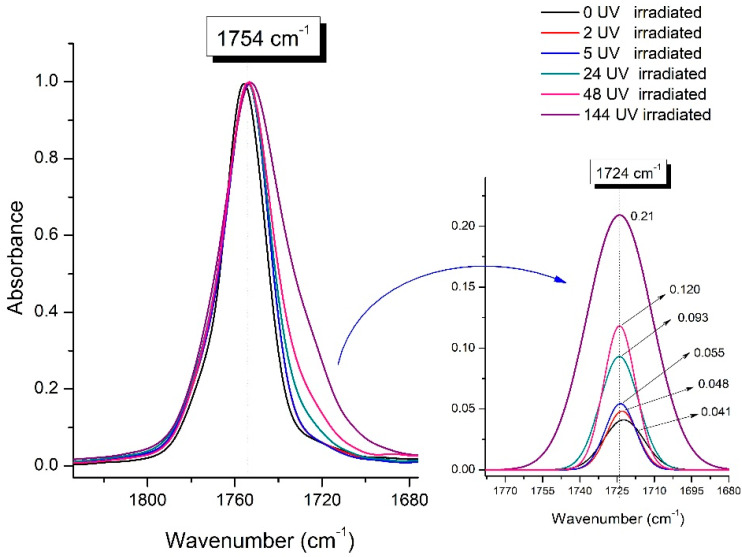
Evolution of the spectral band at 1754 cm^−1^, attributed to the carbonyl group C=O in PLA (left graph); changes in intensity peak at 1724 cm^−1^, belonging to the carbonyl ester group obtained using the regression analysis procedure (right graph).

Figure 9 presents the evolution of the broadening of the main C=O peak at 1754 cm^−1^ (left graph), as well as the change in intensity of the peak at 1724 cm^−1^ (right graph). The data of the peak intensity at 1724 cm^−1^ are presented in Table 4.

Figure 9 (right graph) shows that the intensity of the peak at 1724 cm^−1^ rises as the UV exposure time of PLA increases. At the same time, according to NMR (Figure 6, Supplementary Figures S3 and S4), the basic structure of the polyester skeleton is preserved. In addition, the DSC results show complete amortization of the PLA sample after 144 h of UV exposure (Figure 5, curve 6). Since the NMR and FTIR data of this sample do not indicate qualitative chemical changes in the basic structure of the polymer, this phenomenon may be due to the tautomeric transformations in PLA macromolecules under the UV influence. As a result, some of the units transform from *L*- to the *D*-configuration. Previously, Yasuda et al. [46], with the use of ^13^C NMR analysis, discovered racemization mainly at the terminal units of PLA macromolecules upon UV irradiation.

At the same time, the spatial environment of the carbonyl groups of the ester’s changes is reflected in the FTIR spectrum of the irradiated PLA samples by increasing the peak intensity at 1724 cm^−1^ (Figure 9).

**Figure 10:**
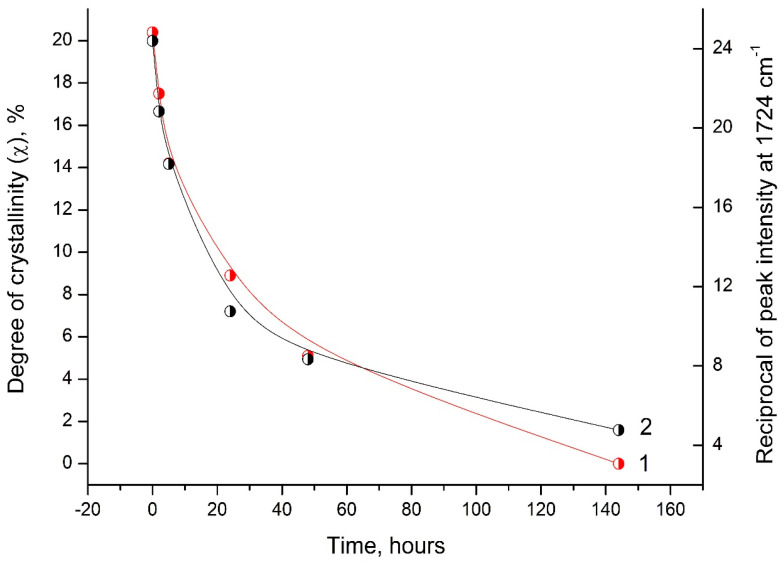
Dependence of the degree of crystallinity (1) and the reciprocal value of the peak intensity at 1724 cm^−1^ (2) on the time of UV irradiation of PLA samples.

Analysis of the DSC and FTIR data indicates a similar behavior of the degree of crystallinity (χ) and the reciprocal value of the intensity peak at 1724 cm^−1^ depending on the time of UV irradiation of the PLA samples (Figure 10).

This correlation between the DSC and FTIR results indicates significant morphological changes in the PLA structure under UV exposure.

## 4. Conclusions

Summarizing all findings obtained by the combination of GPC, NMR, FTIR, and DSC techniques, a significant impact of PLA morphology on its photodegradation has been indicated.

At the initial stage, during the first 5 h, the photolysis of PLA developed intensively in accordance with the Norrish II mechanism of the ester bond cleavage that was supported with GPC data. After this time period, the rate of PLA photodegradation slowed down remarkably. For the first time, this study showed that a distinctive feature of prolonged UV exposure is the emergence of intra- and intermolecular radical recombination reactions, leading to the formation of a high molecular weight fraction of PLA decomposition products.

Along with that, it was found that long-term UV irradiation leads to an increase in the intensity of the absorption stretching vibration band for the carbonyl of the ester groups, the spatial configuration and environment of which differs from the C=O in the original L-PLA. Apparently, its formation is associated with tautomeric transformations in individual polymer units, resulting in the accumulation of *D*-isomeric groups that impede PLA crystallization.

This conclusion was confirmed by the DSC results of a PLA sample irradiated for 144 h, demonstrating its complete amorphization due to the accumulation of D-lactide units preventing polymer crystallization. Analysis of the NMR data enabled the authors to suggest that PLA molecules can be decomposed to form double bonds C=C according to the Norrish II mechanism. Besides, the very low intensity of vinyl protons in the NMR spectrum after 144 h of UV irradiation indicates a Norrish I photodegradation pathway. Moreover, under prolonged UV exposure, the photodegradation of PLA is accompanied by intra- and intermolecular recombination reactions, which depend on the polymer morphology changes.

As a result of the aforementioned, it is reasonable to conclude that PLA photolysis is a complex set of physicochemical processes comprising a combination of Norrish I and II mechanisms as well as the formation of a high molecular weight fraction of PLA decomposition products.

## Figures and Tables

**Table 1:** Values of the weight average mass M_w_, polydispersity PD (M_w_/M_n_), and percentages of the main photodegradation fractions of PLA samples at different UV irradiation times.

UV Irradiation Time for PLA Films (h)	M_w_/M_w_*	PD	Percentage of Main Fractions (M_w_ and M_w_*)
0	132,700	2.4	100
2	46,770	3.3	100
5	34,670	2.3	100
24	12,590/120,220 *	2.1	83.2/16.8 *
144	2818/48,980 *	1.9	76/24 *

Designations marked with * refer to Mw*—average molecular mass of PLA photodegradation products formed during intermolecular recombination of macroradicals.

**Table 2:** DSC parameters of thermal transitions observed in PLA samples exposed to UV irradiation.

Sample	*T*_g_ (°C)	*T*_r_ (°C)	*T*_cc_ (°C)	*T*_m_ (°C)	Δ*H*_cc_ (J/g)	Δ*H*_m_ (J/g)	χ (%)
PLA	63.5	65.1	n/a	162.0–n/a	n/a	−19.1	20.4
PLA UV irradiated for 2 h	57.9	59.7	97.3–82.9 *	161.0–154.2 *	21.2	−37.5	17.5
PLA UV irradiated for 5 h	57.3	59.6	94.7–83.1 *	157.3–148.3 *	28.3	−41.6	14.2
PLA UV irradiated for 24 h	49.4	54.2	92.1–83.5 *	139.3–n/a	11.0	−19.4	8.9
PLA UV irradiated for 48 h	45.0	47.5	n/a	130.5–129.3 *	n/a	−4.8	5.1
PLA UV irradiated for 144 h	35.6	n/a	n/a	n/a	n/a	n/a	−

* refer to the *α*′-limit disordered (hexagonal) crystalline forms of PLA.

**Table 3:** Assignment of bands in the IR spectra of PLA samples after UV irradiation.

Wavenumber (cm^−1^)	Spectral Assignment	Literature
1724	Carbonyl group C=O	[42,43,44]
1454	CH_3_ asymmetric oscillatory mode	[42,43]
1410	This band appears as a result of UV irradiation (OC-OH)	
1382	CH_3_ symmetric strain vibration	[42]
1361	CH deformation and asymmetric bands	[43]
1269	Mixed band; CH bending vibrations and C-CO-C stretching asymmetric vibrations	[42,45]
1211–1184	Asymmetric vibrations of the C-CO-O group and bending vibrations of CH_3_	[42]
1130	CH_3_ asymmetric rocking vibration	[42]
1087	C-O-C symmetric vibrations	[42]
1047	C-CH_3_ stretching vibrations	[42]

**Table 4:** The deconvolution results of peaks in the 1850-1680 cm^−1^ region.

Deconvolution Results Obtained by Regression Analysis Procedure	STATISTICS	Peak Intensity at 1724 (cm^−1^)
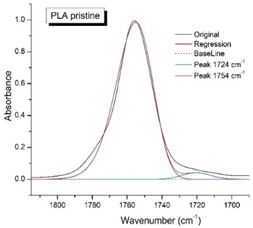	#Correlation coefficient: 0.994785 #Rel.precision: 0.001000 #t-critical(0.95;141): 1.968 #Durbin-Watson Value: 0.102	0.041
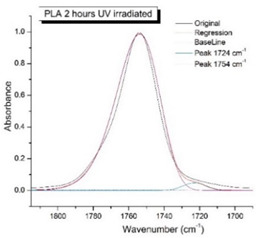	#Correlation coefficient: 0.993925 #Rel.precision: 0.001000 #t-critical(0.95;143): 1.967 #Durbin-Watson Value: 0.084	0.048
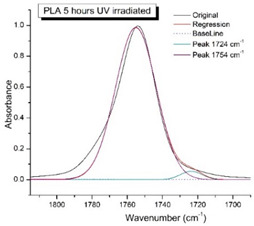	#Correlation coefficient: 0.993082 #Rel.precision: 0.001000 #t-critical(0.95;142): 1.968 #Durbin-Watson Value: 0.078	0.055
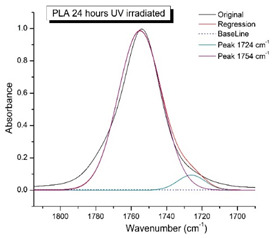	#Correlation coefficient: 0.993316 #Rel.precision: 0.001000 #t-critical(0.95;142): 1.968 #Durbin-Watson Value: 0.066	0.093
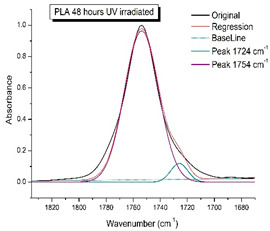	#Correlation coefficient: 0.995271 #Rel.precision: 0.001000 #t-critical(0.95;139): 1.978 #Durbin-Watson Value: 0.077	0.120
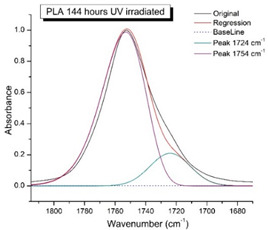	#Correlation coefficient: 0.993947 #Rel.precision: 0.001000 #t-critical(0.95;139): 1.968 #Durbin-Watson Value: 0.032	0.210

## Data Availability

The data presented in this study are available on request from the corresponding author.

## Supplementary Materials

The following supporting information can be downloaded at: https://www.mdpi.com/article/10.3390/polym16070985/s1, Figure S1: Deconvolution results of the ln(M_w_) curves for PLA-24 (a) and PLA-144 (b); Figure S2: Kinetic analysis of PLA photodegradation; Figure S3: ^1^H NMR spectrum of neat PLA (500.18 MHz, CDCl_3_). *δ*, ppm: 1.60 (3H, CH_3_); 5.19 (1H, CH); 4.38 (1H, CH); Figure S4: ^1^H NMR spectrum of PLA after 144 h of UV irradiation (500.18 MHz, CDCl_3_). *δ*, ppm: 1.60 (3H, CH_3_); 5.19 (1H, CH); 4.38 (1H, CH); 5.91, 6.21, 6.52 (CH_2_=CH-); Figure S5: COZY (H, H) spectrum of PLA after 144 h of UV irradiation. *^2^J*_H(a)H(b)_ = 1.5 Hz, *^3^J*_H(b)H(c)_ = 17.0 Hz, and *^3^J*_H(a)H(c)_ = 10.0 Hz.
