# Supplementary material for: Evaluation and Modeling of Polylactide Photodegradation under Ultraviolet Irradiation: Bio-Based Polyester Photolysis Mechanism
Source: Polymers (Basel). 2024 Apr 4;16(7):985. doi: 10.3390/polym16070985 (PMC11013448; doi:10.3390/polym16070985)
Supplement: Supplementary file 1 [file polymers-16-00985-s001.zip › polymers-2918733-supplementary.pdf]

SUPPLEMENTARY MATERIALS

for article

Evaluation and modeling of PLA photodegradation under UV irradiation: Bio-based polyester photolysis mechanism

Sergei Lomakin, Yurii Mikheev, Sergey Usachev, Svetlana Rogovina, Lubov Zhorina, Evgeniya Perepelitsina, Irina Levina, Olga Kuznetsova, Natalia Shilkina, Alexey Iordanskii and Alexander Berlin

The procedure of GPC (Gel Permeation Chromatography) peaks' deconvolution was carried out by means of NETZSCH Peak Separation 2006.01 program employing the nonlinear regression method for asymmetric M_w peaks (Fraser-Suzuki algorithm) [36]. In calculation, the least squares (SLS) reduction was achieved using a hybrid procedure in which the LEVENBERG/MARQUARDT method was combined with step length optimization [J [38].

a b

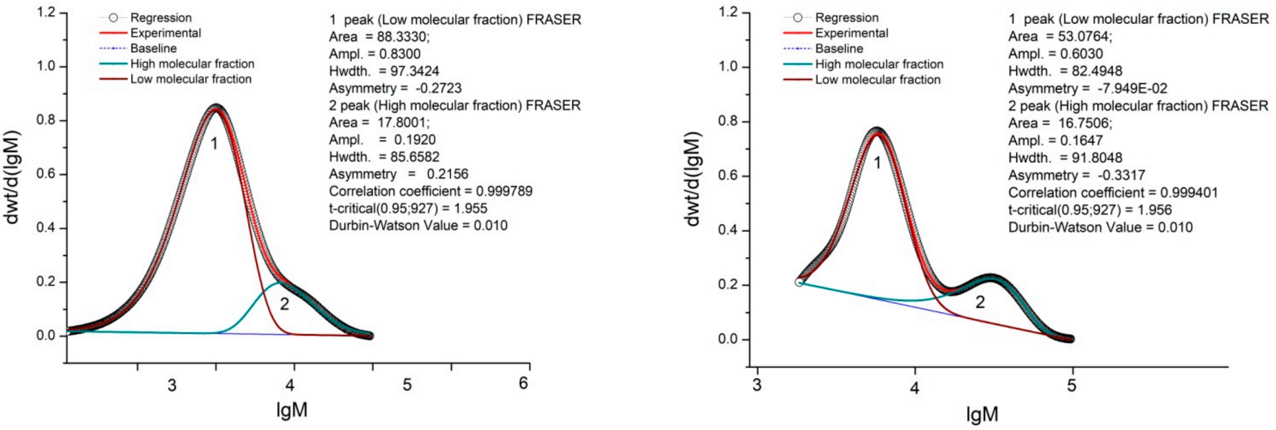

Figure S1. Deconvolution results of the $\ln(M_w)$ curves for PLA-24 (a) and PLA-144 (b).

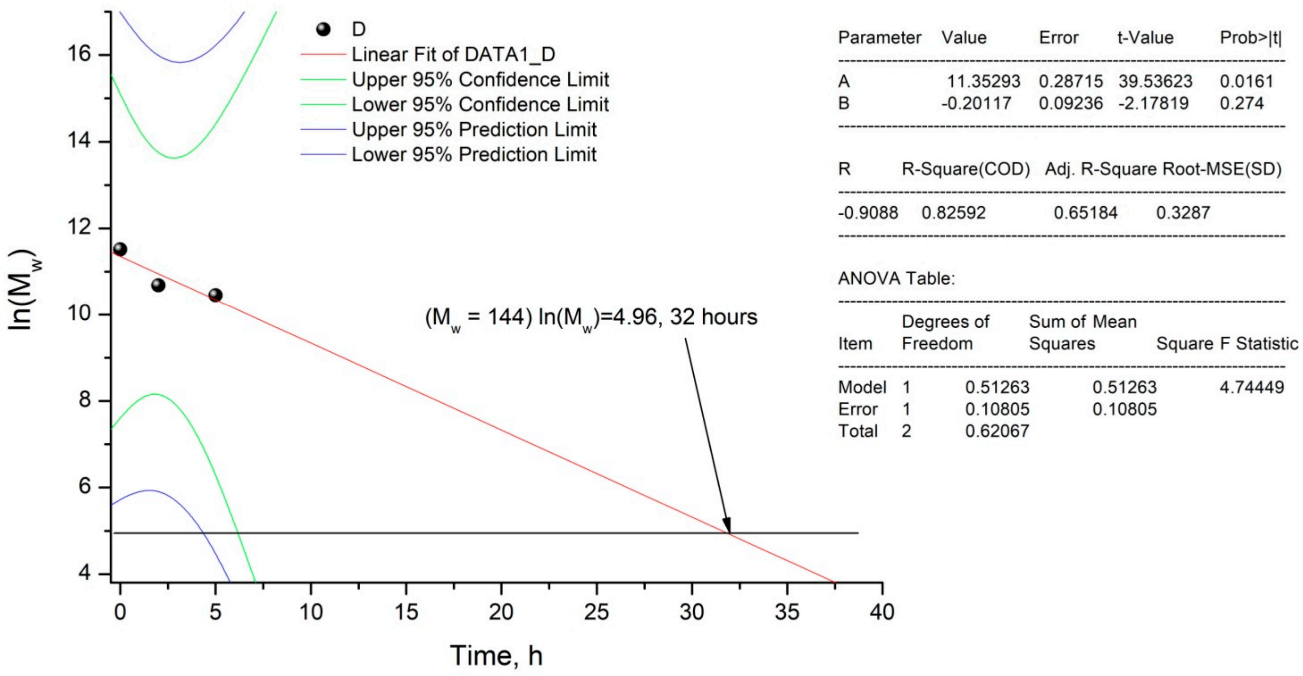

$\ln(M_w) = A + B * \text{time (h)}$

Parameter	Value	Error	t-Value	Prob>|t|
A	11.35293	0.28715	39.53623	0.0161
B	-0.20117	0.09236	-2.17819	0.274

R	R-Square(COD)	Adj. R-Square	Root-MSE(SD)
-0.9088	0.82592	0.65184	0.3287

ANOVA Table:

Item	Degrees of Freedom	Sum of Squares	Mean Square	F Statistic
Model	1	0.51263	0.51263	4.74449
Error	1	0.10805	0.10805	
Total	2	0.62067		

Figure S2. Kinetic analysis of PLA photodegradation.

NMR spectra of initial PLA and PLA after 144 hours of UV irradiation

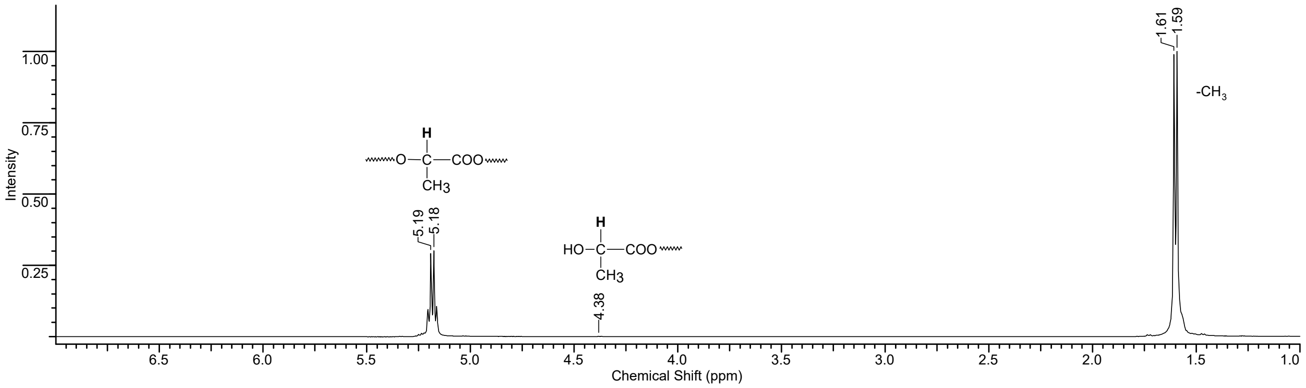

Figure S3. ^{1}H NMR spectrum of neat PLA (500.18 MHz, $CDCl_3$). δ, ppm: 1.60 (3H, CH_3); 5.19 (1H, CH); 4.38 (1H, CH).

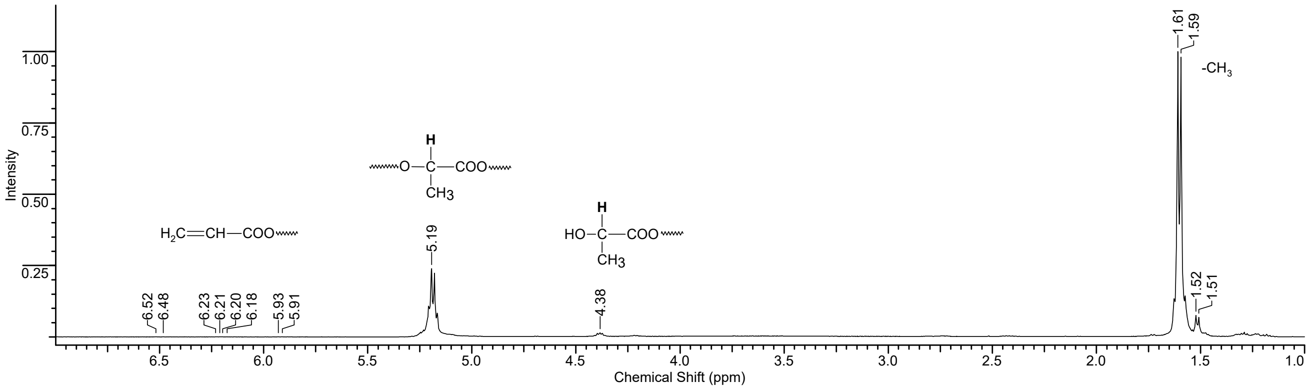

Figure S4. ^{1}H NMR spectrum of PLA after 144 hours of UV irradiation (500.18 MHz, $CDCl_3$).
δ, ppm: 1.60 (3H, CH_3); 5.19 (1H, CH); 4.38 (1H, CH); 5.91, 6.21, 6.52 (CH_2=CH-).

500 MHz (H, H) COZY Spectrum of PLA after 144 hours of UV irradiation

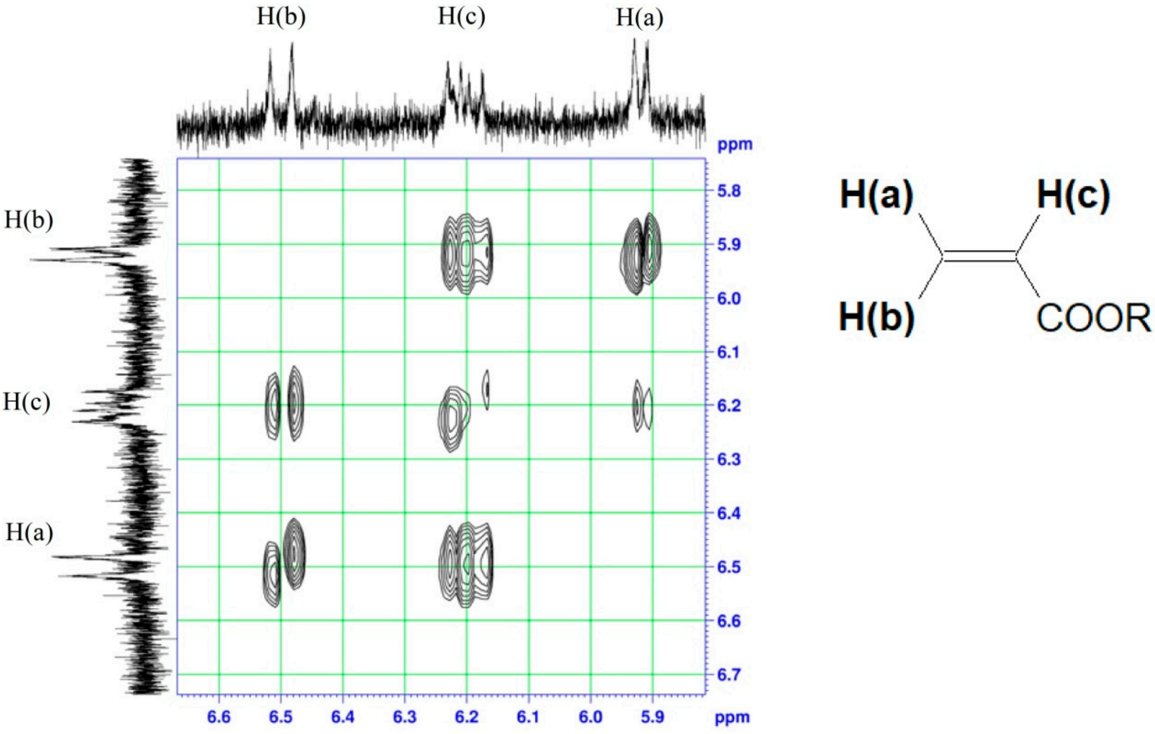

Figure S5. COZY (H, H) spectrum of PLA after 144 hours of UV irradiation. $^{2}J_{H(a)H(b)}$=1.5 Hz, $^{3}J_{H(b)H(c)}$=17.0 Hz and $^{3}J_{H(a)H(c)}$ =10.0 Hz.
